# What Makes Deeply Encoded Items Memorable? Insights into the Levels of Processing Framework from Neuroimaging and Neuromodulation

**DOI:** 10.3389/fpsyt.2014.00061

**Published:** 2014-05-28

**Authors:** Giulia Galli

**Affiliations:** ^1^Brain Investigation and Neuromodulation (BIN) Laboratory, Azienda Ospedaliera Universitaria Senese, Siena, Italy

**Keywords:** levels of processing, episodic memory formation, depth of encoding, TMS, fMRI, tDCS, EEG, psychopharmacology

## Abstract

When we form new memories, their mnestic fate largely depends upon the cognitive operations set in train during encoding. A typical observation in experimental as well as everyday life settings is that if we learn an item using semantic or “deep” operations, such as attending to its meaning, memory will be better than if we learn the same item using more “shallow” operations, such as attending to its structural features. In the psychological literature, this phenomenon has been conceptualized within the “levels of processing” framework and has been consistently replicated since its original proposal by Craik and Lockhart in 1972. However, the exact mechanisms underlying the memory advantage for deeply encoded items are not yet entirely understood. A cognitive neuroscience perspective can add to this field by clarifying the nature of the processes involved in effective deep and shallow encoding and how they are instantiated in the brain, but so far there has been little work to systematically integrate findings from the literature. This work aims to fill this gap by reviewing, first, some of the key neuroimaging findings on the neural correlates of deep and shallow episodic encoding and second, emerging evidence from studies using neuromodulatory approaches such as psychopharmacology and non-invasive brain stimulation. Taken together, these studies help further our understanding of levels of processing. In addition, by showing that deep encoding can be modulated by acting upon specific brain regions or systems, the reviewed studies pave the way for selective enhancements of episodic encoding processes.

## Introduction

Whether we remember an event or not depends on a set of mental processes and brain mechanisms that occur during the initial encoding of the event, its subsequent retrieval, and consolidation processes that take place between encoding and retrieval. Among the factors that act upon encoding, the level to which an item is cognitively processed largely affects memorability. This levels of processing (LOP) framework was originally proposed by Craik and Lockhart in 1972 (1), and has since then fueled debate in episodic memory research. In a typical experiment, depth is manipulated by asking participants to engage deep or shallow processing on the to-be-remembered items during encoding (2). For instance, judging whether a word represents a living or a non-living entity is a deep encoding task because it requires semantic analysis and access to the meaning of the word. By contrast, judging whether a word contains a given letter is a shallow encoding task as it only requires structural and phonological analysis. Other shallow encoding tasks, such as syllable, rhyme, or pleasantness judgments, involve an intermediate level of analysis along the structural–semantic axis. Typically, items encoded using semantic operations are better remembered in a subsequent memory test than items encoded using shallow operations at any level of depth (2). LOP effects affect later performance even in the absence of any deliberate intention to learn, and are in fact most frequently studied using unintentional encoding. The superiority of memory performance after deep encoding is not only one of the most robust findings in episodic memory research, but it is also clearly recognizable by most experimental participants, and both these factors contribute to the intuitive appeal of the LOP framework.

In general, theorists agree that deep encoding results in more elaborate memory traces, and that this in turn affects later memorability. But what exactly constitutes an elaborate memory trace, and what are the mechanisms that make elaborate memory traces more memorable? The psychological literature has emphasized that enhanced distinctiveness and integration with pre-existing knowledge are among the factors that contribute to the memory benefit for items that received deep processing at encoding (3, 4). Yet, it is not entirely clear what are the exact mechanisms underlying LOP effects, and how they are instantiated in the brain. Some authors argue that neuroimaging does not help explain the “experience of memory” and that the debate on LOP effects should remain within the boundaries of experimental psychology (5). However, there are a number of relevant questions that a cognitive neuroscience perspective can help address. For instance, knowing which brain regions are activated by deep and shallow encoding and how these activations relate to subsequent memory may inform on the specific processes at play during the two types of encoding, and on the nature of the differences (e.g., qualitative vs. quantitative) between them. Psychopharmacological or non-invasive brain stimulation (NIBS) interventions may add insights into what modulates neural activity associated with deep and shallow encoding in these brain regions, and the relative contribution of encoding and retrieval operations to LOP effects.

This work draws from the cognitive neuroscience literature to describe, first, some of the key neuroimaging findings in this field, and the main questions related to the LOP framework that have been addressed in the past few years of research. I will focus on investigations of neural activity associated with successful episodic memory encoding, therefore on those studies that analyzed neural activity associated with deep and shallow processing at encoding as a function of later memory performance. Clearly, whether a given item will be remembered or not also depends on a set of processes that are specific to the retrieval situation, such as the way memory is probed, and the similarity between encoding and retrieval contexts (6, 7). However, an examination of retrieval-specific mechanisms is beyond the aims of the current review, and brain activations associated with retrieval success and recognition memory judgments will not be discussed. I will then review recent findings from CNS-active psychopharmacological interventions that helped clarifying the nature of processes involved in deep and shallow encoding. Finally, I will discuss how NIBS holds promise for future studies aiming at investigating LOP effects, and maybe pave the way for selective enhancements of episodic encoding processes.

## Neural Correlates of Effective Deep and Shallow Encoding

Since the advent of event-related neuroimaging, several studies have investigated the neural correlates of successful memory formation, which involves a comparison of brain activity at encoding, separately for items that are remembered or forgotten in a subsequent memory test. The rationale for this subsequent memory procedure (8) is that determining the neurobiological processes that influence whether an event will be memorable is of vital importance for the understanding of episodic memory. Functional magnetic resonance imaging (fMRI) investigations have consistently reported subsequent memory effects in the ventral and dorsal prefrontal cortex (PFC), medial temporal lobe (MTL), including the hippocampus and parahippocampal regions, and parietal cortex [for reviews see Ref. (9–12)]. In human electrophysiological studies, successful memory formation is indexed by positive-going event-related potentials (ERPs) recorded over anterior scalp sites, and a complex pattern of brain oscillations [for reviews see Ref. (13, 14)]. Only a few studies however (15–24) investigated differences in neural activity associated with deep and shallow encoding tasks as a function of subsequent memory performance.

Most of these studies aimed to investigate whether deep and shallow processing leading to successful encoding differ qualitatively or quantitatively. In other words, whether they are expression of distinct mnemonic mechanisms, or, rather, different levels or strengths of a single encoding mechanism. In terms of brain substrates, this translates into the question of whether episodic encoding relies on a single neural system irrespective of encoding task, or it is supported by multiple, task-specific systems. As a general standpoint, this question is complex because it requires a precise separation between deep and shallow encoding, which is in practice hard to achieve. The answer is, indeed, not easy. On the one hand, a good number of studies have demonstrated that a largely similar set of brain regions is implicated in successful deep and shallow encoding (15, 16, 19, 20). More specifically, these studies have shown that the brain regions associated with shallow encoding are a subset of those engaged in deep encoding, with no brain region uniquely associated with the former (15, 16, 19). For instance, Otten et al. (19) demonstrated that remembered words that were deeply studied showed fMRI activations in bilateral inferior frontal gyrus and left anterior and posterior hippocampus, while words encoded in the shallow task elicited subsequent memory effects only in the anterior hippocampus and in a smaller portion of the left inferior frontal gyrus. Evidence of a quantitative, rather than qualitative, difference between deep and shallow subsequent memory effects was also demonstrated in ERPs and magnetoencephalography studies (21, 22, 25). These findings may suggest that memory formation relies on a single neural system, irrespective of the encoding task.

In contrast with this view, two fMRI studies (17, 24) found subsequent memory effects specific for shallow encoding in posterior brain regions, involving the bilateral posterior sulcus, bilateral fusiform gyrus, and left occipital gyrus (17), and an increased functional connectivity between the right hippocampus and the right DLPFC-parietal network (24). However, it should be noted that both Otten and Rugg (17) and Schott et al. (24) used a syllable judgment encoding task (reporting the number of syllables that compose a word). This task, while admittedly shallow, is at an intermediate level of depth compared to the alphabetic task used in the studies reviewed so far. In addition, syllable judgments involve processes that rely on posterior brain regions, such as counting or inferring the number of syllables from the length of the word (26), with only limited engagement of the left PFC (27). Subsequent memory effects in parietal areas associated with a syllable judgment encoding task have indeed been reported before (28). It thus appears that memory formation for syllable judgments involves specific brain regions, which support the online encoding task, whereas other shallow encoding tasks, such as alphabetical judgments, may engage brain activations in prefrontal areas (29), and therefore overlap with those associated with deep encoding. This leads to another relevant question addressed by neuroimaging research, which is central for the understanding of the mechanisms underlying LOP effects, that is, the overlap between task-specific and subsequent memory related activations.

Neuroimaging studies have consistently shown that task- or stimulus-specific brain regions activated during encoding (e.g., areas selectively activated by semantic and structural processing, or by a specific class of stimuli, such as faces) also demonstrated subsequent memory effects (15–17, 19, 21, 30). For instance, the signal increase associated with the deep encoding task in left inferior prefrontal and MTL regions mirrored the signal increase that emerged in the subsequent memory contrast for deeply encoded items within the same regions (15–17, 19, 21, 24). Notably, a recent functional connectivity study found increased connectivity between the left PFC and the hippocampus associated with both the semantic task and subsequent memory for deeply encoded stimuli (24). Analogous results in posterior brain regions were demonstrated for shallow encoding (17, 24). These findings crucially suggest that memory formation engages the activation of a subset of brain regions that support online, task-specific processing. In other words, effective episodic encoding is supported by products of the processing engaged by the encoding task.

One hypothesis is that during deep encoding, semantic elaboration supported by the left inferior PFC (27, 31, 32) automatically activates pre-existing knowledge and semantically associated information about the item, perhaps through a temporary semantic working memory system (15, 17, 32). The subsequent memory effects in the left inferior PFC and functional connectivity with the hippocampus observed for deep encoding may thus reflect the benefits of incorporating these semantic associations with the studied item into a unique representation of the study episode (10). In other words, during deep encoding items are bound to the contextual aspects of the study episode, which is one of the key components to form a coherent episode in memory (33). It is reasonable to assume that this mechanism is at least in part responsible for the superior memory performance, but also for the higher proportion of confident and recollection-based responses, associated with deep encoding (34). In contrast, shallow encoding tasks that heavily rely on structural processing, such as judging whether a word contains the letter “E,” do not engender a sufficiently deep level of analysis to allow associative and contextual processes unfold, and therefore the engagement of relational processes and MTL structures would only be minimal. In between semantic and structural encoding tasks, episodic records for syllable judgments could perhaps incorporate some information derived from the encoding task, such as the word length. This could be reflected in increased functional connectivity between the PFC and the hippocampus for shallow encoding (24).

Taken together, the neuroimaging findings reviewed so far complement and extend previous knowledge on LOP effects, and on episodic memory in general. They suggest that effective deep and shallow encoding may be qualitatively or quantitatively different, depending on the specific processes that are active during the encoding task, and substantiate the idea that the episodic memory of an event is a byproduct of these processes (35). Task-specific and relational processing at encoding, associated with corresponding brain activations, may be ways in which memory formation for deeply encoded items is enhanced.

## Selective Modulation of Memory for Deeply Encoded Events: Evidence from Psychopharmacological Studies

Episodic memory is modulated by a number of neurotransmitters and CNS-active drugs. Studies that investigated the effects of pharmacological interventions on LOP vary with respect to the pharmacokinetic and pharmacodynamic properties of the drug, the dose, and time of administration with respect to the memory phase (encoding or retrieval), and the memory test used to probe memory. That said, there is sufficient commonality in the studies to allow some comparison and integration.

One of the most widely studied neurotransmission systems in relation to memory is the neocortical cholinergic system. Acetylcholine (ACh) projects from the basal forebrain to the cortex and the hippocampus, which contains one of the highest densities of cholinergic terminals and receptors (36). The PFC also shows dense cholinergic innervation (37). Given the key role of these brain structures in learning and memory (9, 10, 12), the modulation of memory functions by ACh is not surprising. Although the effect of pro-cholinergic drugs is not consistent across studies on healthy young and elderly participants (38, 39), acetylcholinesterase inhibitors enhance episodic memory performance in patients with Alzheimer’s disease (40), and are routine symptomatic treatments for memory decline in this clinical condition.

A few studies have investigated behavioral and brain activation patterns associated with LOP effects following administration of drugs acting on cholinergic pathways, namely the acetylcholinesterase inhibitors Donepezil and physostigmine (30, 41), and nicotine (42). In all these studies, memory accuracy increased following drug administration. In addition, the cholinergic neuromodulation interacted with LOP at encoding, as the memory enhancement was restricted to deeply studied stimuli, while leaving memory accuracy for shallowly encoded items unaffected. This may appear surprising at a first glance – ultimately, deeply encoded items should be less vulnerable to modulations as they involve stronger memory traces. So why would cholinergic effects act upon deeply, but not shallowly, encoded items? A recent fMRI study by Bentley and colleagues (30) offers a plausible explanation. In this study, elderly individuals and Alzheimer’s patients received physostigmine or placebo during deep and shallow encoding of images depicting faces or buildings. Volunteers had to judge whether a particular face or building was old or young in the deep encoding task, or whether the image was red or green in the shallow encoding task. For face stimuli, the results showed that in elderly individuals physostigmine increased subsequent memory performance for deep, but not shallowly encoded items. This behavioral advantage was associated with increased activations during deep encoding in the face-selective fusiform cortex, and with increased functional coupling between the fusiform cortex and the right hippocampus. In contrast, in Alzheimer’s patients physostigmine did not induce task-dependent behavioral or brain activation changes. These findings substantiate the neuroimaging findings reviewed in the previous section by showing that effective deep encoding is supported by the activation of online, task- or stimulus-specific areas, and by their connections with MTL structures. They further extend previous evidence suggesting that the cholinergic system could be a crucial mediator of this effect. One caveat of the cholinergic studies reviewed so far is that the effect of the drug covered both encoding and retrieval. Given the well-known diverging effects of pro-cholinergic drugs on encoding and retrieval operations (43), future studies could investigate whether the interaction between LOP effects and ACh is dependent upon the time of administration with respect to the memory phase.

Whereas ACh generally facilitates episodic memory, other neurotransmitter systems are associated with reduced memorability. Ketamine, an antagonist of the *N*-methyl-d-aspartate (NMDA) receptor, and inhibitory neurotransmitters of the gamma-aminobutyric acid (GABA) system, such as benzodiazepines, induce drastic decreases in memory performance (44–46). For instance, the facilitation of GABA inhibits the functioning of the hippocampus, inducing dose-related decrements in episodic memory (47). At the neural level, the memory impairment is accompanied by encoding-related deactivations following benzodiazepine administration in the left dorsal PFC (48), left inferior PFC, and hippocampus (49). The modulation of memory performance and brain activations following ketamine and benzodiazepine administration is probably dependent upon the dense concentration of their receptor sites in the hippocampus and cerebral cortex (50, 51).

With respect to LOP, the effects of drugs with sedative and amnesic effects have been fairly inconsistent. Lorazepam and ketamine administration was associated with decrements of recognition memory accuracy, selectively for deeply encoded items, or items with an intermediate level of depth (52–54). However, studies from the same groups using similar doses and procedures showed no interaction between drug effects and LOP (45, 55, 56). It is not clear how to reconcile these diverging findings. Nevertheless, it is worth noticing that similar to the effects of ACh, the effects of ketamine, and benzodiazepines, if any, act upon deep but not shallow encoding. One could speculate that because of the dense populations of NMDA and benzodiazepine receptors in the frontal cortex and hippocampus, and the extensive recruitment of these brain structures in deep encoding, it is more likely that any disruption would affect deep encoding to a larger extent. Interestingly, Honey et al. (45) demonstrated that following ketamine administration brain activity in the left ventrolateral PFC associated with a deep, compared to a shallow, encoding task increased. This suggests that ketamine may selectively affect task-specific processing that supports successful memory formation. Investigations using other drugs with sedative actions produced additional divergent results, with no interaction between drug and LOP [barbiturates: Ref. (57)], and again selective impairment for deeply encoded items [anesthetic propofol: Ref. (58, 59)].

Finally, and surprisingly given its strong influence on memory (60), cortisol does not seem to interact with LOP. However, the effects of cortisol largely vary depending on dose, timing of administration relative to the memory phase, time of the day of testing, emotional content of the stimulus, and arousal state at the time of testing (61, 62). The relation between cortisol and memory is thus very complex, and future studies may find an effect of cortisol on LOP when controlling for some of these variables.

The body of work summarized here suggests that neurotransmitter systems such as cholinergic, GABA-ergic, and NMDA systems have a non-generic sedation or enhancing effect on episodic memory. Perhaps because of their pattern of receptor innervation in the brain, ketamine, ACh, and benzodiazepines selectively affect the memorability of items encoded using deep operations. The modulation of neural activity in brain regions that support the online encoding task may be one way in which CNS-active drugs act upon memory formation of elaborate memory traces. This discussion emphasizes the need of further research on the specific mechanisms that contribute to drug-induced improvements or decrements of episodic memory.

## Neuromodulation of Depth of Processing by Non-Invasive Brain Stimulation: Emerging Evidence

Functional magnetic resonance imaging and electrophysiological techniques are inherently correlational, therefore, it is not possible on the basis of their data alone to determine whether neural activity is necessary to a specific task. NIBS techniques instead can provide information on the causal role of a specific brain region in a given cognitive process. Transcranial magnetic stimulation (TMS) and transcranial direct current stimulation (tDCS) are the most widely adopted NIBS techniques in the investigation of memory functions.

Transcranial magnetic stimulation uses a magnetic field to induce changes in the resting potentials of the underlying cortex and thus in its electrical currents. This determines a transient interruption of the normal brain activity and interference with cognitive processing (63). TMS can be delivered as a single pulse, or as a series of single pulses (repetitive TMS, rTMS), and can have facilitatory or inhibitory effects depending on the frequency of stimulation. In contrast, tDCS delivers constant, low-intensity (up to 2 mA), electrical currents to the scalp via two large anode and cathode electrodes (64). The current modifies resting membrane potentials and the spontaneous firing rate of neurons in a polarity-dependent fashion, without however inducing action potentials (65). Because of their distinct physiological mechanisms, TMS and tDCS differ in the type of information they provide. TMS stimulation is focal, whereas the spatial resolution of tDCS is limited. In addition, TMS is generally locked in time with stimulus presentation, or other events of interest. The temporal dynamics of the engagement of a given brain region can thus be identified by observing the effects of TMS in this region at different points in time (66–69). In contrast, tDCS is not locked to the presentation of single events, rather it is delivered over a prolonged period of time off-line or during the task.

To investigate memory formation, TMS or tDCS is typically delivered over the target area and one or more control sites during encoding, and subsequent memory performance is then assessed as a function of the stimulation condition. On the whole, NIBS studies confirmed previous fMRI and PET evidence of the key role of the PFC in episodic memory formation, either in the dorsal (68, 70–78) or ventral (66, 67, 79–82) portions. It is worth remembering that as the depth of stimulation is limited to a few centimeters, TMS and tDCS cannot directly stimulate some of the key regions involved in episodic memory formation, such as MTL structures. However, a recent neuroimaging study (83) has shown that the stimulation can modulate intrinsic brain network dynamics and propagate to distal brain structures, including the hippocampus.

The majority of brain stimulation studies only adopted one encoding strategy, consisting of a semantic judgment. To date, only two TMS studies directly compared deep and shallow encoding tasks and their effect on memory (76, 84). Innocenti et al. (76) delivered 10 Hz rTMS to the left and right dorsolateral PFC during a semantic and an alphabetical judgment encoding task. The effect of the stimulations on subsequent memory performance was compared with the stimulation of a control site and a no-TMS condition (baseline). Consistent with previous studies (68, 70–75), rTMS delivered over the left dorsolateral PFC decreased recognition accuracy compared to the other stimulation conditions. However, this effect was specific to semantically encoded words. In the study by Vidal-Piñeiro et al. (84) instead, memory for deep and shallow encoding was equally unaffected by the off-line theta burst stimulation (TBS) of a more ventral region of the PFC. However, as evidenced by a post-TMS fMRI scan, TBS increased activations of the left ventrolateral PFC, occipital cortex, and cerebellum, and the connectivity between these brain regions, while volunteers were performing the deep encoding task. These findings suggest that the combination of neuroimaging and brain stimulation offers relevant insights into the brain networks involved in LOP effects, even in the absence of overt behavioral effects.

There are several methodological differences between these two TMS studies that may have determined the discrepancy of behavioral effects, including differences in the protocol and site of stimulation. Nevertheless, once again the literature offers a scenario in which the neural or behavioral modulation is specific to semantic encoding. Along the same vein of what has been suggested for psychopharmacological studies, neuromodulation with TMS may interfere with task-specific and associative processes that support the online semantic task. Unfortunately, performance for the semantic encoding task is generally at ceiling, and this makes it hard to detect any effect of neuromodulation at encoding. In fact, investigations that used a neuromodulatory approach either did not report performance data for the encoding tasks, or reported a lack of effects.

In summary, TMS holds promise for future investigations of LOP effects. The possibility to selectively interfere with specific stages of memory (encoding vs. retrieval) makes this technique an excellent candidate for the study of the relative role of encoding and retrieval operations in determining LOP effects. Further studies using different stimulation protocols, sites, and timings are needed to expand our knowledge of selective effects of TMS on deep encoding. Electrical stimulation with tDCS could also further our understanding of LOP effects. For instance, the differential effects of anodal and cathodal tDCS on episodic memory performance (85) may induce dissociations in LOP effects, thereby adding to the investigation of the nature of the differences between deep and shallow encoding. In addition, given that anodal tDCS induces enhancements in episodic memory performance in healthy young and elderly individuals (78), it will be of great interest in future studies to assess whether tDCS can selectively induce memory enhancements according to the depth of encoding. This could be relevant especially for those pathological conditions that are characterized by memory impairments of deep but not shallow encoding (86–88). Finally, the observation that subsequent memory for deep and shallow encoding is associated with different patterns of oscillatory brain activity (23, 89, 90), will provide impetus for the investigation of the effects of rhythmic brain stimulation (rTMS and transcranial alternating current stimulation) on depth of processing.

## Conclusion

In this article, I reviewed the contribution of neuroimaging, psychopharmacological, and NIBS studies to our understanding of LOP effects. Taken together, the findings discussed here provide partial answers to the question of “what makes deeply encoded items more memorable?” They suggest that memory formation for deeply encoded events is enhanced when the products of online, task-specific processing are integrated with pre-existing knowledge about the event into a coherent episodic memory trace. At the neural level, this is reflected in overlapping task- and encoding-related brain activations, and their functional connections with MTL structures. These findings therefore converge with the psychological literature, which has previously suggested that the episodic memory of an event is a byproduct of the processes active during encoding (35), and that the integration with pre-existing knowledge contributes to the memory benefit for items that receive deep processing at encoding (3, 4). Crucially, these cognitive and neural processes are mediated by activity in cholinergic, GABA-ergic, and NMDA neurotransmitter systems, which analogously to NIBS, specifically modulate memory formation for deep encoding. The proposed mechanism may not be exclusive to deep encoding *per se*. Rather, it may generalize to shallow encoding task that are of sufficient depth to induce associative process in the formation of the episodic record.

It is important to note that a process-based account need not be the only explanation for the specificity of the effects for deep encoding. For instance, the number of trials for shallow encoding in any given subsequent memory comparison is generally small due to low memory performance. Therefore, the power to detect any statistical difference in this condition is low. One could speculate that distinct subsequent memory patterns would emerge if a shallow encoding task that yields higher memory accuracy was used. In this view, the posterior subsequent memory effects for shallow encoding reported in Otten and Rugg (17) and Schott et al. (24) could be attributable to the higher number of trials in the syllable judgment encoding task, rather than to the specific processes involved in this task. Distinguishing between these alternative views will be difficult, but future studies could address this issue through careful examinations of how systematic variations of encoding tasks yielding different levels of memory accuracy correspond to linear changes in brain activation patterns.

Finally, one should emphasize that the mechanisms enhancing memory formation for deeply encoded events reviewed here provide only part of what is needed to accurately remember those events, that is, they provide the *potential* of retrieval (4). Equally important are the processes that occur during retrieval, the way memory is later tested, and the overlap of the encoding and retrieval situation (6, 7). The possibility to selectively interfere with different stages of the memory process make neuromodulatory approaches excellent candidates for the investigation of the interdependence of encoding and retrieval operations.

## Conflict of Interest Statement

The author declares that the research was conducted in the absence of any commercial or financial relationships that could be construed as a potential conflict of interest.
